
JOURNAL INFORMATION
==============================
NLM Title Abbreviation: Access Microbiol
Journal ID: Access Microbiol
NLM Journal ID: 101746927
Journal ID: acmi

Access Microbiology

EISSN: 2516-8290
Publisher: Microbiology Society

ARTICLE INFORMATION
==============================
PMCID: PMC7491927
PMID: 32974495
Article version: 1
Subjects: Oral Abstract

Abstracts from the Antimicrobial Drug Discovery from Traditional and Historical Medicine Meeting 2019

Publication date: 2019
Electronic publication date: 2019 Dec 20
Volume: 1
Issue: 10
Electronic Location ID: 3
Copyright: © 2019 The Authors
Copyright year: 2019
License: This is an open-access article distributed under the terms of the Creative Commons Attribution License.
License URL: https://creativecommons.org/licenses/by/4.0/

==============================


Antimicrobial properties from lichens: An evaluation of the antimicrobial properties of English churchyard lichens

http://jmmcr.microbiologyresearch.org

Taylor Judith *
Fourie Toscane
The Open University , Milton Keynes , United Kingdom
Institute de recherche biomedicale des armees , Aix-Marseille , France
*Correspondence:Judith Taylor, judith.taylor@open.ac.uk

Understanding extraction principles underpinning historical antimicrobial drug discovery for improving rediscovery and reproducibility

http://jmmcr.microbiologyresearch.org

Keulen Geertje van *
Luft Diana
Jenkins Rowena E.
Institute of Life Science , Swansea University Medical School , Swansea
Centre for Advanced Welsh & Celtic Studies , University of Wales , Aberystwyth
*Correspondence:Geertje van Keulen, g.van.keulen@swansea.ac.uk

Developing high through put screening strategies to identify the next generation of antimicrobials

http://jmmcr.microbiologyresearch.org

Booth Peter
McCarthy Ronan *
Brunel University London , London , United Kingdom
*Correspondence:Ronan McCarthy, ronan.mccarthy@brunel.ac.uk

Deciphering the biological activity of propolis (bee glue) and its constituents; what is its potential as an antimicrobial?

http://jmmcr.microbiologyresearch.org

Escalada Margarita Gomez *
Leeds Beckett University , Leeds , United Kingdom
*Correspondence:Margarita Gomez Escalada, m.gomez-escalada@leedsbeckett.ac.uk

A strong inhibitory effect of heather honey, propolis and medicinal plant extracts on biofilm formation by pathogenic bacteria

http://jmmcr.microbiologyresearch.org

Billah Zara
Attar Baraa
Hughes Lisa
Seidel Veronique
Efthimiou Georgios *
University of Strathclyde , Glasgow , United Kingdom
University of Hull , Hull , United Kingdom
*Correspondence:Georgios Efthimiou, miou80@yahoo.com

An investigation into the anti-microbial properties of bacterial cellulose wound dressings loaded with curcumin:hydroxypropyl-β-cyclodextrin supramolecular inclusion complex

http://jmmcr.microbiologyresearch.org

Swingler Sam † *
Gupta Abhishek †
Heaselgrave Wayne
Kowalczuk Marek
Radecka Iza *
University of Wolverhampton , Wolverhampton , United Kingdom
Research Institute in Healthcare Science, FSE, University of Wolverhampton , Wolverhampton , United Kingdom
Polish Academy of Sciences , Zabrze , Poland
*Correspondence:Sam Swingler, S.Swingler@wlv.ac.uk*Correspondence:Iza Radecka, I.Radecka@wlv.ac.uk

† These authors contributed equally to this work.

